# Current Molecular Epidemiology of Methicillin-Resistant *Staphylococcus aureus* in Elderly French People: Troublesome Clones on the Horizon

**DOI:** 10.3389/fmicb.2016.00031

**Published:** 2016-01-28

**Authors:** Claire Rondeau, Guillaume Chevet, Dominique S. Blanc, Houssein Gbaguidi-Haore, Marie Decalonne, Sandra Dos Santos, Roland Quentin, Nathalie van der Mee-Marquet

**Affiliations:** ^1^UMR 1282, Réseau des Hygiénistes du Centre, Centre Hospitalier Universitaire de ToursTours, France; ^2^Service of Hospital Preventive Medicine, Lausanne University HospitalLausanne, Switzerland; ^3^Service d’Hygiène Hospitalière, Centre Hospitalier Universitaire de BesançonBesançon, France; ^4^Département de Bactériologie et Hygiène, Centre Hospitalier Universitaire de ToursTours, France

**Keywords:** *Staphylococcus aureus* MRSA, elderly, carriage, bloodstream infection, qacA/B

## Abstract

**Objective:** In 2015, we conducted at 44 healthcare facilities (HCFs) and 21 nursing homes (NHs) a 3-month bloodstream infection (BSI) survey, and a 1-day prevalence study to determine the rate of carriage of methicillin-resistant *Staphylococcus aureus* (MRSA) in 891 patients and 470 residents. We investigated the molecular characteristics of the BSI-associated and colonizing MRSA isolates, and assessed cross-transmission using double-locus sequence typing and pulsed-field gel electrophoresis protocol.

**Results:** The incidence of MRSA-BSI was 0.040/1000 patient-days (19 cases). The prevalence of MRSA carriage was 4.2% in patients (*n* = 39) and 8.7% in residents (*n* = 41) (*p* < 0.001). BSI-associated and colonizing isolates were similar: none were PVL-positive; 86.9% belonged to clonal complexes 5 and 8; 93.9% were resistant to fluoroquinolones. The *qacA/B* gene was carried by 15.8% of the BSI-associated isolates [3/3 BSI cases in intensive care units (ICUs)], and 7.7% of the colonizing isolates in HCFs. Probable resident-to-resident transmission was identified in four NHs.

**Conclusion:** Despite generally reassuring results, we identified two key concerns. First, a worryingly high prevalence of the *qacA/B* gene in MRSA isolates. Antisepsis measures being crucial to prevent healthcare-associated infections, our findings raise questions about the potential risk associated with chlorhexidine use in *qac*A/B^+^ MRSA carriers, particularly in ICUs. Second, NHs are a weak link in MRSA control. MRSA spread was not controlled at several NHs; because of their frequent contact with the community, conditions are favorable for these NHs to serve as reservoirs of USA300 clone for local HCFs.

## Introduction

Methicillin-resistant *Staphylococcus aureus* (MRSA) infections have long been associated with healthcare facilities (HCFs) and remain a matter of concern, due to the morbidity and mortality associated with the infections they cause (de Kraker et al., 2011). Over the last 20 years, the adherence of healthcare workers to infection control guidelines for patients with MRSA carriage has improved. This approach has proved effective, leading to a marked decrease in the incidence of healthcare-associated infections (HAIs) due to MRSA (Cooper et al., 2004; McGinigle et al., 2008; Rolain et al., 2015).

However, the epidemiology of MRSA is changing, with the worldwide spread of Panton-Valentine leukocidin (PVL)-producing MRSA previously implicated only in infections in patients with no history of healthcare (Boyce, 2008; Popovich et al., 2008). The spread of the ST8-USA300 clone is a matter of particular concern. These MRSA isolates are increasingly being implicated in HAIs (Tenover and Goering, 2009). In France, infections associated with PVL-producing MRSA remain rare (Robert et al., 2011). However, two clusters of ST8-USA300 infections were recently described, one in a rehabilitation care center (Fournier, 2013) and the other in a residential home (Haut Conseil de la Santé Publique, 2014).

We investigated epidemiological changes in the large “Centre Val de Loire” region (2.6 million inhabitants), assessing MRSA infection and colonization in the elderly, by conducting a multicenter study. We conducted a 3-month survey of bloodstream infections (BSI) associated with MRSA, and a 1-day prevalence study of MRSA carriage in patients and residents. We studied the molecular characteristics of the BSI-associated and colonizing MRSA isolates, and assessed cross-transmission within institutions using molecular typing tools. In addition, chlorhexidine resistance in MRSA being an emerging threat (Edgeworth, 2011), the MRSA isolates were screened for the presence of the *qacA/B* gene, i.e., the genetic determinant for eﬄux-mediated resistance to chlorhexidine (Poole, 2005).

## Materials and Methods

### Survey of Bloodstream Infection

A 3-month survey of all cases of BSI was conducted at 47 HCFs (45 hospitals and two home hospitalization centers) and nine independent nursing homes (NHs) between January 1 and March 31 2015. These institutions had a total of 7,381 short-stay beds, 2,187 rehabilitation care beds and 8,549 long-stay beds. The survey covered 473,600 patient-days (PDs) in short-stay units, 134,428 PDs in rehabilitation care units and 70,966 PDs in long-stay units.

The methods used and the design of this study have been reported elsewhere (van der Mee-Marquet et al., 2007). Briefly, BSI was defined as a positive blood culture from a patient with clinical or laboratory evidence of infection. The variables reported included patient age and sex, origin of the BSI and occurrence of death within 7 days of BSI diagnosis. All *S. aureus* isolates from BSI cases were sent to the central laboratory for extensive study. Identification was confirmed by MALDI-TOF MS (bioMérieux, France). Antimicrobial drug susceptibility testing was performed by the agar disk diffusion method (Comité de l’Antibiogramme de la Société Française de Microbiologie, 2015). The antibiotics tested were penicillin G, oxacillin, erythromycin, lincomycin, pristinamycin, tetracycline, kanamycin, tobramycin, gentamicin, rifampin, fusidic acid, fosfomycin, pefloxacin and cotrimoxazole. Vancomycin, teicoplanin and mupirocin Minimum Inhibitory Concentration values were determined using E-test method (bioMérieux, France). PCR assays were used to confirm the presence of the *mec*A gene (Cuny et al., 2011) and to detect the *luk*S-*PV* and *lukF-PV* genes (Jarraud et al., 2002) and the genetic determinant for chlorhexidine resistance *qacA/B* (Fritz et al., 2013). MRSA isolates were typed using pulsed-field gel electrophoresis protocol (Murchan et al., 2003), and double-locus sequence typing (DLST) (Basset et al., 2010). DLST-types were assigned according to the DLST database (www.DLST.org). Using eBURST algorithm, the relatedness between isolates was defined by clustering all DLST-types sharing one of the two alleles.

### Carriage Study

The study was conducted at the 56 institutions participating in the BSI study. The HCFs had medical, surgical and rehabilitation care units, and at least one NH unit. For the 45 HCFs, we first selected one unit at random. We then selected 30 individuals from this unit at random, for participation in the study. We selected 30 individuals at random from each of the independent NHs and home hospitalization centers. All the patients and residents present in the institutions between January and March 2015 were eligible for recruitment. They (or their relatives) were approached individually and asked for consent to access their medical records and to culture a sample to test for *S. aureus*.

The 1-day point-prevalence study was carried out between January 1 and March 31 2015. Each patient or resident was included only once. Microbiological samples were collected from all the individuals included. Patients and residents were screened for *S. aureus* carriage by nasal swabbing (both nostrils). The swabs were placed in 0.5 ml of sterile water and 0.1 ml of the resulting suspension was streaked onto a Chapman selective agar plate (bioMérieux, France). The plates were incubated for 48 h at 35°C. The identification and molecular characterization of *S. aureus* and MRSA isolates were as described for the BSI study.

Data were collected on a standardized questionnaire, for all the individuals enrolled in the study. They included demographic data (age and sex), physical disability and significant comorbidities, and various risk factors for MRSA carriage (hospitalization and antibiotic use in the month preceding inclusion in the study).

Univariate analyses were carried out with χ^2^ or Fisher’s exact tests, as appropriate, for categorical variables and Student’s *t*-test or Kruskall–Wallis test for continuous variables. All statistical tests were two-tailed. We considered *p*-values <0.05 to be statistically significant. We used EpiInfo version 6 software and Stata software version 10 (StataCorp., USA) for statistical analysis.

#### Confidentiality and Ethical Aspects

The study was carried out in accordance with French recommendations for the prevention of infection in healthcare. Ethical approval was obtained at the national level, from the *Réseau Alerte Investigation Surveillance des Infections Nosocomiales* (RAISIN). The study was managed jointly with the directors of the HCFs and NHs, the hygiene nurses and the physicians responsible for caring for the patients/residents, and the regional infection control team.

## Results

### BSI Study

#### Epidemiology

Nineteen cases of BSI due to MRSA were identified, 12 in male patients and seven in female patients hospitalized in short-stay units, including three in intensive care units (ICUs). This resulted in an incidence rate of 0.040/1,000 PDs. The BSI was associated with endocarditis (*n* = 1), infections of the skin (*n* = 5), lungs (*n* = 4), the urinary tract (*n* = 3), a surgical site (*n* = 2) or an intravenous device (*n* = 4). Most of the patients had a recent history of hospitalization (63.2%), and of antibiotic treatment (42.1%); one patient lived in a NH (5.3%). The early mortality rate was 21.0%.

#### Molecular Characteristics of the BSI Isolates

**FIGURE 1:**
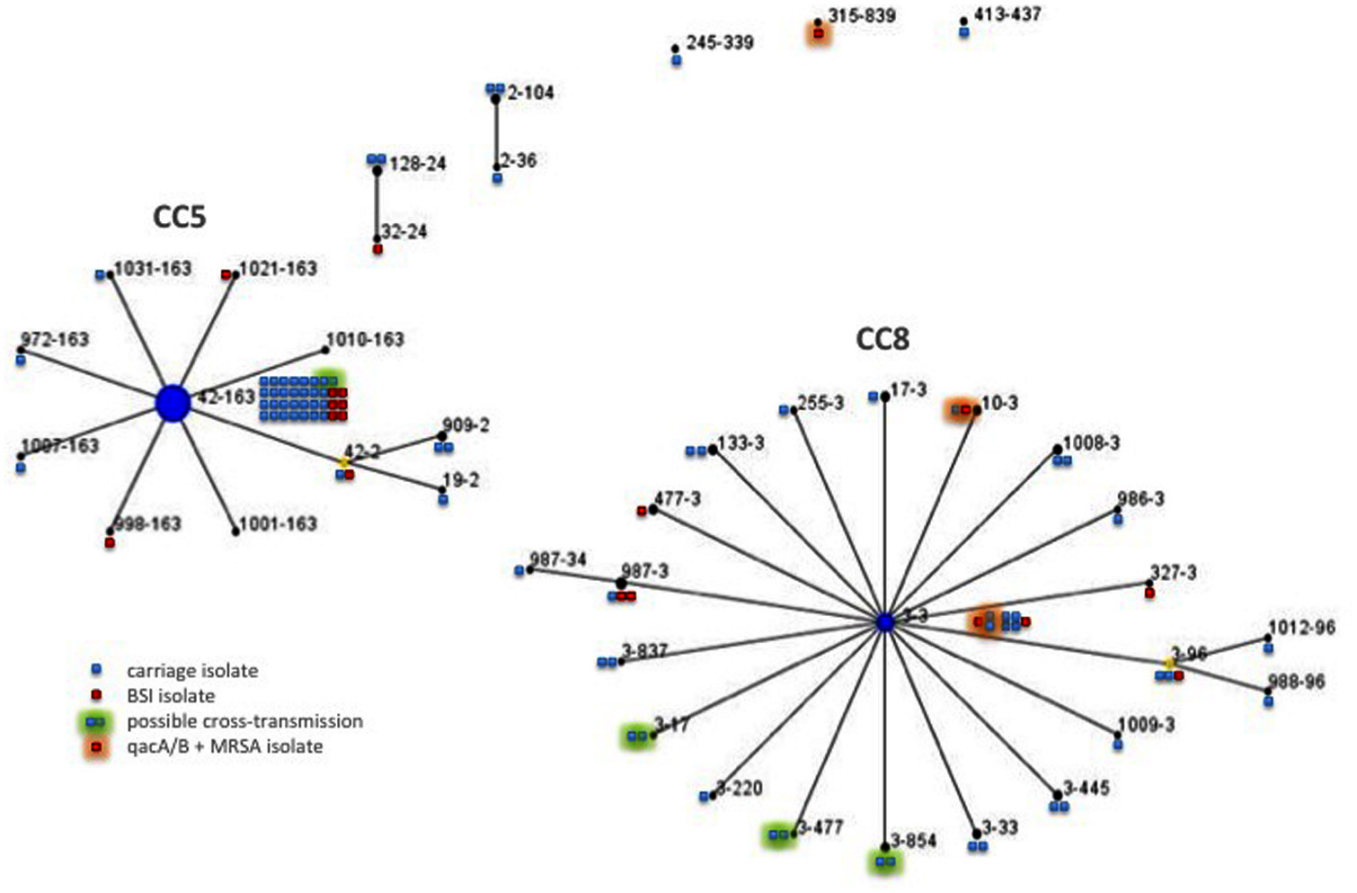
**Double locus sequence typing (DLST) single locus variant (SLV) clustering using eBurst on the 19 BSI-associated and 80 colonizing MRSA isolates**. Each circle represents one DLST-type and the number of the squares reflects the frequency of that type among colonizing (in blue) or BSI-associated isolates (in red). Each green halo indicates possible epidemiologically related isolates and black halo indicates isolates carrying *qac*A/B gene. Linked DLST-type differ at one of the two loci (*clfB* or *spa*).

The PVL gene was not detected in the 19 isolates studied. All but one of the MRSA isolates were resistant to fluoroquinolones (94.7%) (Supplementary Table S1); 12 were resistant only to methicillin and fluoroquinolones (63.2%). Eburst clustering revealed two major clusters: one comprising 9 (47.5%) isolates from clonal complex (CC) 5 and the other containing 8 isolates from CC8 (42.1%) (**Figure 1**). The *qacA/B* gene was detected in three isolates (15.8%) recovered from the three patients hospitalized in different ICUs (Supplementary Table S1, **Figure 1**). One had a BSI of surgical origin, and the other two had BSI of pulmonary origin, developing during the course of mechanical ventilation. For these two cases, chlorhexidine mouth washes were carried out daily during hospitalization.

### Carriage Study

#### Population Characteristics

Sixty-five units participated in the study (**Table 1**); 921 patients and 470 residents were enrolled in the study: 819 women (58.9%) and 572 men (41.1%). The population was elderly (median age: 82 years) and had a poor health status, with 40.3% of individuals having a McCabe index of 1 or 2 (fatal illness likely to occur within the next 5 years). Cancer and immunodepression were reported in 10.4 and 8.1% of the study subjects, respectively. A recent history of urinary catheterization or of the insertion of an intravenous device was reported for 4.9 and 14.7% of the subjects studied, respectively. In addition, 14.4% of the individuals had recently been treated with antibiotics, and, 13.6% presented signs of infection on the day of the study. The clinical characteristics differed significantly between patients and residents. The very elderly population of residents had a better health status, with significantly fewer comorbid conditions than the population of patients (**Table 1**). Among patients, those hospitalized in surgical units were younger (*p* < 0.001) and had a better health status than those from medical and rehabilitation care units.

**Table 1:** Demographic, clinical characteristics and MRSA carriage of the 921 patients and 470 residents enrolled in the MRSA carriage study.

	Healthcare facilities medical unit	Surgical unit	Rehab.^1^	Other units^2^	Nursing homes
Participating units	11	9	20	4	21
Patients/residents	354	280	794	229	1276
Patients/residents enrolled	206	185	452	78	470
Median age in years (gender)	85 (F) 82 (M)	73 (F) 69 (M)	82 (F) 75 (M)	64 (F) 69 (M)	88 (F) 83 (M)
McCabe index 1–2 (%)	51.6	13.0	37.1	68.9	41.9
Cancer (%)	13.2	4.4	13.6	19.5	7.1
Immunodepression (%)	9.7	2.2	10.5	37.7	2.6
Recent history of	10.2	7.1	4.5	10.7	1.5
urinary catheterization (%)					
Intravenous device use (%)	36.4	38.6	6.4	14.7	3.6
Antibiotic treatment (%)	43.2	14.6	13.3	8.0	5.1
Signs of infection on the day of the study (%)	41.0	10.4	12.6	4.0	5.5
*Staphylococcus aureus* carriers (%)	41 (19.9)	44 (23.8)	108 (23.9)	20 (25.6)	130 (25.7)
MSSA carriers (%)	30 (14.6)	43 (23.2)	85 (18.8)	16 (20.5)	89 (18.9)
MRSA carriers (%)	11 (5.3)	1 (0.5)	23 (5.1)	4 (5.1)	41 (8.7)

*^1^Rehabilitation unit. ^2^One dialysis unit, two home hospitalization centers, one psychiatry unit*.

#### MRSA Carriage

*Staphylococcus aureus* carriage was found in 213 patients (23.1%) and 130 residents (27.6%); 39 patients (4.2%) and 41 residents (8.7%) were found to be MRSA carriers. The carriage rate was between 0 and 25.0%, depending on the unit considered, and was higher in residents than in patients (*p* < 0.001) (**Table 2**). A carriage rate >10.0% was observed in 15.9% of the HCF units and in 42.9% of the NH units (*p* < 0.018). Among patients, the MRSA carriage rate was lower in surgical units (*p* = 0.005). Twelve of the 80 MRSA carriers (15.0%) presented signs of infection on the day of the study, but staphylococcal infection was unlikely in all the cases. MRSA carriers were older than the patients not carrying MRSA (*p* = 0.003) and they also had a poorer health status: MRSA carriers were more likely to have a McCabe index of 1 or 2 (*p* = 0.017), cancer (*p* = 0.037) or an infection (*p* = 0.030) (**Table 2**). Among residents, MRSA colonization was associated with poorer health status (*p* = 0.022) and recent antibiotic treatment (*p* = 0.013) (**Table 2**). The residents and patients did not differ in terms of clinical characteristics, except for median age (*p* = 0.006).

**Table 2:** Characteristics of the 921 patients and 470 residents enrolled in the MRSA carriage study, by MRSA carriage status.

Healthcare facilities	MRSA carriers	MSSA carriers	*S.aureus* non-carriers	MRSA non-carriers	*p^∗^*
Number of patients	39	174	708	882	
Median age (years)	83	76	79	79	0.034
McCabe index 1–2 (%)	59.0	38.5	34.2	38.6	0.017
Cancer (%)	23.1	11.5	11.4	11.6	0.037
Immunodepression (%)	18.4	10.3	10.4	10.6	
Recent history of urinary catheterization (%)	10.2	4.0	7.1	6.5	
urinary catheterization (%)					
Intravenous device use (%)	25.6	23.0	22.3	20.0	
Antibiotic treatment (%)	28.2	12.1	19.9	18.9	
Signs of infection on the day of the study (%)	30.8	10.3	18.4	17.2	0.030

**Nursing homes**

Number of residents	41	89	340	429	
Median age (years)	87	88	86	86	
McCabe index 1–2 (%)	51.2	35.9	36.8	33.5	0.022
Cancer (%)	7.5	6.7	7.1	7.1	
Immunodepression (%)	7.5	3.4	1.8	2.1	0.075
Recent history of	4.9	2.2	0.1	1.2	
urinary catheterization (%)					
Intravenous device use (%)	0	3.4	4.1	3.4	
Antibiotic treatment (%)	14.6	1.1	5.0	4.2	0.013
Signs of infection on the day of the study (%)	12.2	1.1	5.9	4.9	0.066

*Comparison of MRSA carriers and MRSA non-carriers; only significant *p*-values are shown.

#### Molecular Characteristics of Colonizing MRSA Isolates

The PVL gene was not detected in the 80 isolates studied. Most of the 80 isolates were resistant to fluoroquinolones (93.7%; Supplementary Table S1). Fifty-one isolates (63.8%) were resistant only to methicillin and fluoroquinolones. DLST of the 80 isolates identified 34 DLST-types (**Figure 1**). Like the BSI isolates, most colonizing isolates formed two major clusters, one comprising 35 (43.7%) isolates from CC5 and the other containing 34 isolates from CC8 (42.5%). The isolates colonizing residents were associated with CC5 whereas those from patients were associated with CC8 (*p* = 0.044). The *qacA/B* gene was detected in three of the 39 isolates recovered from patients (7.7%) (Supplementary Table S1, **Figure 1**); high level of resistance to mupirocin (MIC value > 1024 mg/L) was observed in one of these 39 isolates (2.6%) (Chaves et al., 2004). Isolates carrying the *qacA/B* gene or mupirocin resistant were not recovered from residents.

#### Spread of MRSA Within Institutions

In 12 cases, two or more isolates of the same DLST-type were recovered from individuals from the same unit (Supplementary Table S2). In four NH units, the isolates with similar DLST-types also yielded similar PFGE patterns, suggesting an epidemiological link between the isolates and cross-transmission within the NH. The rate of MRSA carriage at these four NH units exceeded 11.0%. Separation of the 65 participating units into two groups on the basis of carriage rate (below or above 10%) revealed an association between possible cross-transmission and the group of units with the highest carriage rates (*p* = 0.003).

## Discussion

Outside of the context of PVL-producing MRSA spread, data concerning MRSA in elderly are scarce. We report a large-scale multicenter study of MRSA BSI and carriage in elderly people and provide insight into the current epidemiology of MRSA. Our findings were generally reassuring.

Consistent with results of other studies (March et al., 2009; Köck et al., 2010; Bellini et al., 2015), we found a low incidence of MRSA-BSI in our region, an overall MRSA carriage rate of 4.2% in patients and 8.7% in residents, and a significant link between MRSA BSI or carriage and very old individuals with numerous comorbid conditions, recent antibiotic exposure and infection.

Concordant with previous studies, colonizing and infecting isolates were genetically similar (Robinson and Enright, 2003; Safdar and Bradley, 2008), and the isolates were equally distributed among the major pandemic CCs CC5 and CC8 (Eko et al., 2015). MRSA belonging to CC8 were mostly resistant to fluoroquinolones, erythromycin and aminoglycosides, while most of isolates of CC5 were only resistant to fluoroquinolones. DLST analysis indicated wide heterogeneity among the studied isolates, apart from a particular clone characterized by a unique DLST-type (i.e., 42–163) and clustering one third of the MRSA isolates recovered from colonized and infected patients/residents hospitalized into highly diverse HCIs and NHs. These findings suggest a successful clone spreading into our region.

We detected no colonizing MRSA isolates from the USA300 clone in the nasal flora of the 1391 subjects enrolled. Despite the limitations of our study design, such as the use of a single nasal screening (McKinnell et al., 2013; Warnke et al., 2014), our findings suggest that the spread of this clone has not yet significantly affected the institutions in our region, whereas clusters of infections associated with this clone have recently been detected in France (Fournier, 2013; Haut Conseil de la Santé Publique, 2014). However, we identified two key concerns.

### A Worryingly High Prevalence of the *qac*A/B Gene in MRSA Isolates

The application of “search and isolate” or “search and destroy” policies for MRSA carriers in ICUs, and the isolation of patients infected with MRSA in all HCFs have resulted in a decrease in the incidence of infections caused by MRSA (McDanel et al., 2013). In this context, chlorhexidine is increasingly used, especially as an antiseptic for skin and mucosal membranes in HCFs, and for daily mouth wash in ICUs (Milstone et al., 2008; Batra et al., 2010). Bacterial survival following exposure to antiseptics has been recognized and mechanisms conferring decreased susceptibility to antiseptics have been identified (Harbarth et al., 2014). Recent studies have described unintended consequences following the use of chlorhexidine. First, the presence of the *qacA/B* gene has been associated with unsuccessful decolonization during an ICU-based topical chlorhexidine intervention (Lee et al., 2011); second, the prevalence of reduced chlorhexidine susceptibility has been shown to be high in organisms causing central line-associated BSIs in ICUs in which patients are bathed daily with chlorhexidine (Suwantarat et al., 2014); and third, a MRSA isolate that carried the *qacA/B* gene and had chlorhexidine MBC threefold higher compared with other strains circulating on the unit, was selected following the introduction of a chlorhexidine-based decolonization regimen (Batra et al., 2010). In addition, several studies have underlined how the presence of *qacA/B* gene may confer a selective advantage in the presence of chlorhexidine use (Ho and Branley, 2012; Otter et al., 2013). Before this study, data about the presence of the *qacA/B* gene in MRSA isolates colonizing/infecting elderly French people were not available. The high prevalence of the *qacA/B* gene in BSI isolates (3/3 cases in ICUs), and in isolates into the microbiota of patients is worrisome. These aspects require further prospective investigation and continued surveillance. To date, there is a lack of a standard definition of resistance to antiseptics, absence of standardized methods of detection, and no systematic method for measuring the clinical impact of reduced susceptibility to antiseptics (Harbarth et al., 2014). Our findings raise questions about the relationship between the carriage of chlorhexidine eﬄux-mediated resistance genes and reduced chlorhexidine susceptibility, and about the potential risk associated with chlorhexidine use in *qacA/B*^+^ MRSA carriers, particularly in ICUs. Reduced susceptibility to antiseptics could become an increasing problem; its clinical impact needs further research.

### NHs Remain a Weak Link in MRSA Control

The relatively low rates of MRSA carriage masked different situations: rates ranged from 0 to 25.0% in participating units, whereas the prevalence of comorbid conditions and risk factors were similar for all units and could not account for the observed differences. The spread of MRSA was controlled in most participating NH units. Whereas this occurred without the application of a “search and isolate” strategy for MRSA carriers in these NHs. This indirectly confirm that in NHs, where invasive care procedures are rare and generally concern residents in reasonable health, the application of standard precautions during care procedures and the respect of basic hand hygiene measures by all staff and, as far as possible, by the residents, prevent the resident-to-resident transmission of MRSA.

Colonizing isolates carried by more than one resident yielded similar DLST types in several NHs, suggestive of intra-NH resident-to-resident transmission. However, the design of our study does not allow to various other possibilities to be excluded, for example, importation from acute care settings that cared the many of the residents. In HCFs, the colonizing isolates were genetically diverse, probably reflecting better respect of hygiene rules in HCFs, whereas standard precautions remain inconsistently applied in NHs (Cochard et al., 2014). In NHs, the risk of infection is low and infections are rare. Consequently, the care staff does not apply hygiene practices rigorously when caring for the residents and between cares acts for different residents, and MRSA may spread on their hands. In addition, unlike patients confined to bed in HCFs, residents frequently interact with other residents, thereby spreading MRSA via their hands if their hand hygiene is not of quality. In situations in which residents are repeatedly hospitalized, the NHs may serve as reservoirs of MRSA for the local HCFs. Because of their frequent contact with the community in which the USA300 clone is spreading, the NHs could serve as a site for the spread of this troublesome clone in the near future in the absence of significant improvements in preventive measures.

This argues for continual efforts to prevent the spread of MRSA in NHs. Education is required to promote hand hygiene, the appropriate use of gloves and the restriction of glove use when not required. Point-prevalence studies of carriage should be conducted regularly in NHs, and MRSA carriage rates should be used as a quality indicator to focus the attention of healthcare workers on the spread of MRSA.

## Author Contributions

All authors listed have made substantial contribution to the work (CR, GC, MD, and SDS: acquisition of data; NVDM, DB, and HG: data analysis and interpretation of data, NVDM, and RQ: study design and drafting the paper), and approved it for publication.

## Conflict of Interest Statement

The authors declare that the research was conducted in the absence of any commercial or financial relationships that could be construed as a potential conflict of interest.
