# Supplementary material for: Current Molecular Epidemiology of Methicillin-Resistant Staphylococcus aureus in Elderly French People: Troublesome Clones on the Horizon
Source: Front Microbiol. 2016 Jan 28;7:31. doi: 10.3389/fmicb.2016.00031 (PMC4729942; doi:10.3389/fmicb.2016.00031)
Supplement: Supplementary file 1 [file Table_1.DOCX]

**Supplementary table 1.**

Antibiotic susceptibility pattern of the 80 colonizing MRSA isolates and the 19 bloodstream infection-associated MRSA isolates

| Antibiotype* | Nb | Colonizing MRSA | | BSI-associated MRSA |
| --- | --- | --- | --- | --- |
|  | of isolates | HCF | NH |  |
| FQ | 51 **^(1)^ | 21 **^(1)^ | 30 | 12 |
| KT FQ | 5 | 3 | 2 |  |
| KT FQ (L) | 1 |  | 1 |  |
| KT L FQ | 3 ** |  |  | 3 **^(1)^ |
| KT FQ FA MUP | 1 | 1 |  |  |
| KT EL FQ | 6 **^(4)^ | 4 **^(2)^ |  | 2 **^(2)^ |
| KT E | 1 | 1 |  |  |
| FQ (L) | 4 | 2 | 2 |  |
| EL FQ | 3 | 2 |  | 1 |
| E FQ | 3 | 2 | 1 |  |
| T FQ | 1 | 1 |  |  |
| FA | 1 |  | 1 |  |
| FQ TE | 1 |  | 1 |  |
| TE | 1 | 1 |  |  |
| TE FQ L | 1 |  | 1 |  |
| T FA | 1 |  | 1 |  |
| FQ FA | 1 | 1 |  |  |
| - | 2 |  | 1 | 1 |
| ALL | 99**^(6)^ | 39**^(3)^ | 41 | 19**^(3)^ |

* FQ pefloxacin, K kanamycin, T tobramycin, E erythromycin, L lincomycin, FA fusidic acid, MUP mupirocin, TE tetracycline

**(*n*) presence of the *qacA/B* gene in *n* isolates
