# Supplementary material for: Current Molecular Epidemiology of Methicillin-Resistant Staphylococcus aureus in Elderly French People: Troublesome Clones on the Horizon
Source: Front Microbiol. 2016 Jan 28;7:31. doi: 10.3389/fmicb.2016.00031 (PMC4729942; doi:10.3389/fmicb.2016.00031)
Supplement: Supplementary file 2 [file Table_2.DOCX]

**Supplementary table 2**.

Molecular typing results for the colonizing MRSA isolates recovered from the same HCF/NH and with a similar DLST type

| Healthcare facility/  nursing home | Participating unit | Number of  MRSA carriers  (carriage rate) | DLST type  (nb of isolates with  same DLST-type) | PFGE pattern of isolates with similar DLST types |
| --- | --- | --- | --- | --- |

| hci-447 | rc-447 | 2 (8.0) | 3-133 (2) | 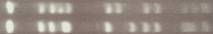 | related |
| --- | --- | --- | --- | --- | --- |
|  |  |  |  |  |  |

| nh-3202 (hci-132) | nhu-3202 | 2 (8.0) | 163-42 (2) | 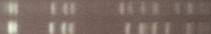 | related |
| --- | --- | --- | --- | --- | --- |
|  |  |  |  |  |  |
| nh-3079 | nhu-3079 | 3 (11.1) | 3-17 (2) | 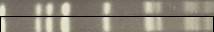 | identical |
|  |  |  |  |  |  |
| nh-3364 (hci-115) | nhu-3364 | 3 (15.0) | 163-42 (2) | 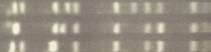 | related |
|  |  |  |  |  |  |
| nh-2769 | nhu-2769 | 3 (15.8) | 2-909 (2) | 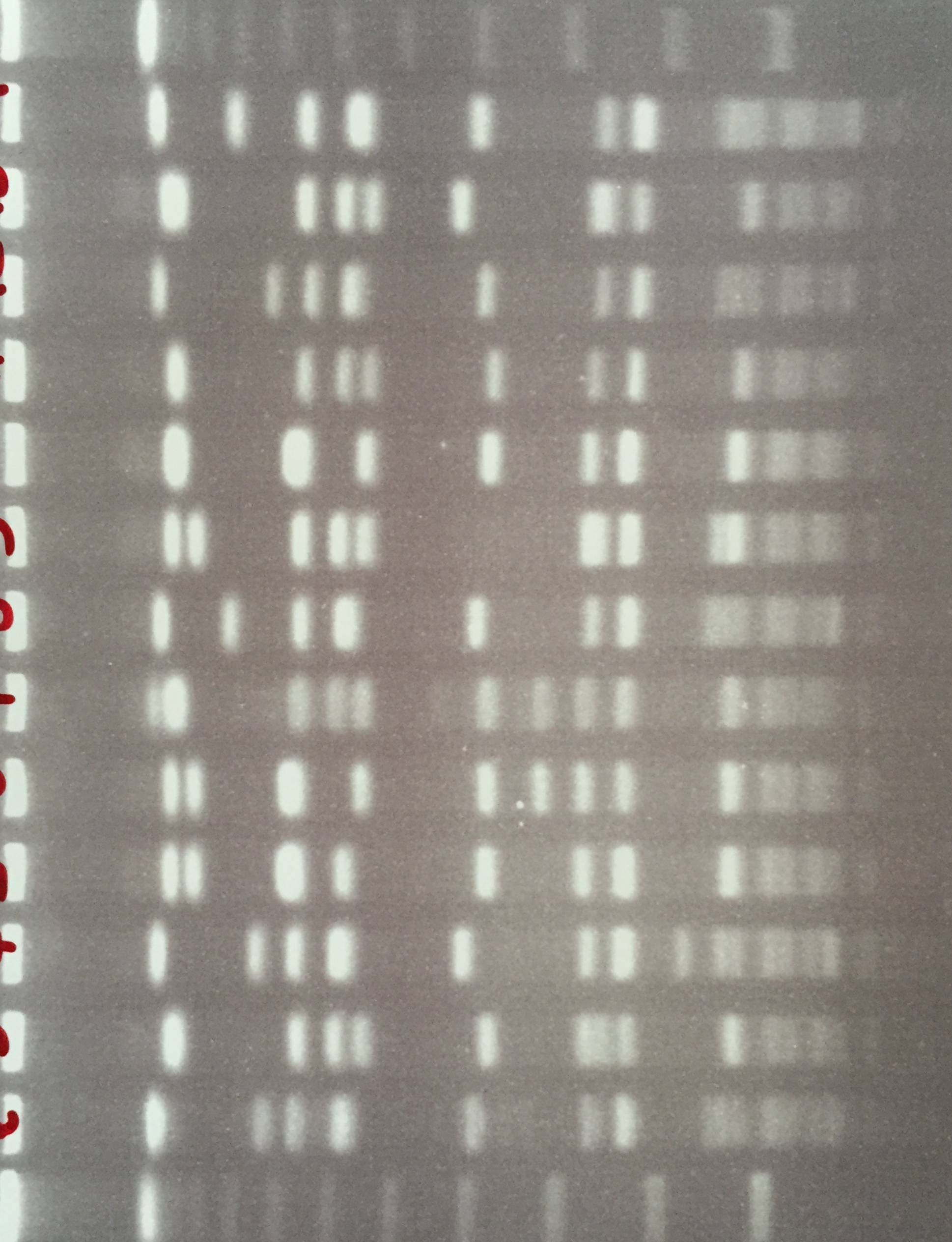 | related |
|  |  |  |  |  |  |
| nh-3167 (hci-125) | nhu-3167 | 4 (19.0) | 163-42 (2)  3-477 (2) | 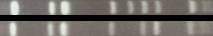  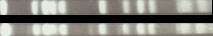 | related  identical |
|  |  |  |  |  |  |
| nh-3234 (hci-631) | nhu-3234 | 3 (20.0) | 163-42 (2) | 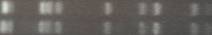 | identical |
|  |  |  |  |  |  |
| nh-3110 (hci-119) | nhu-3110 | 4 (20.0) | 163-42 (2) | 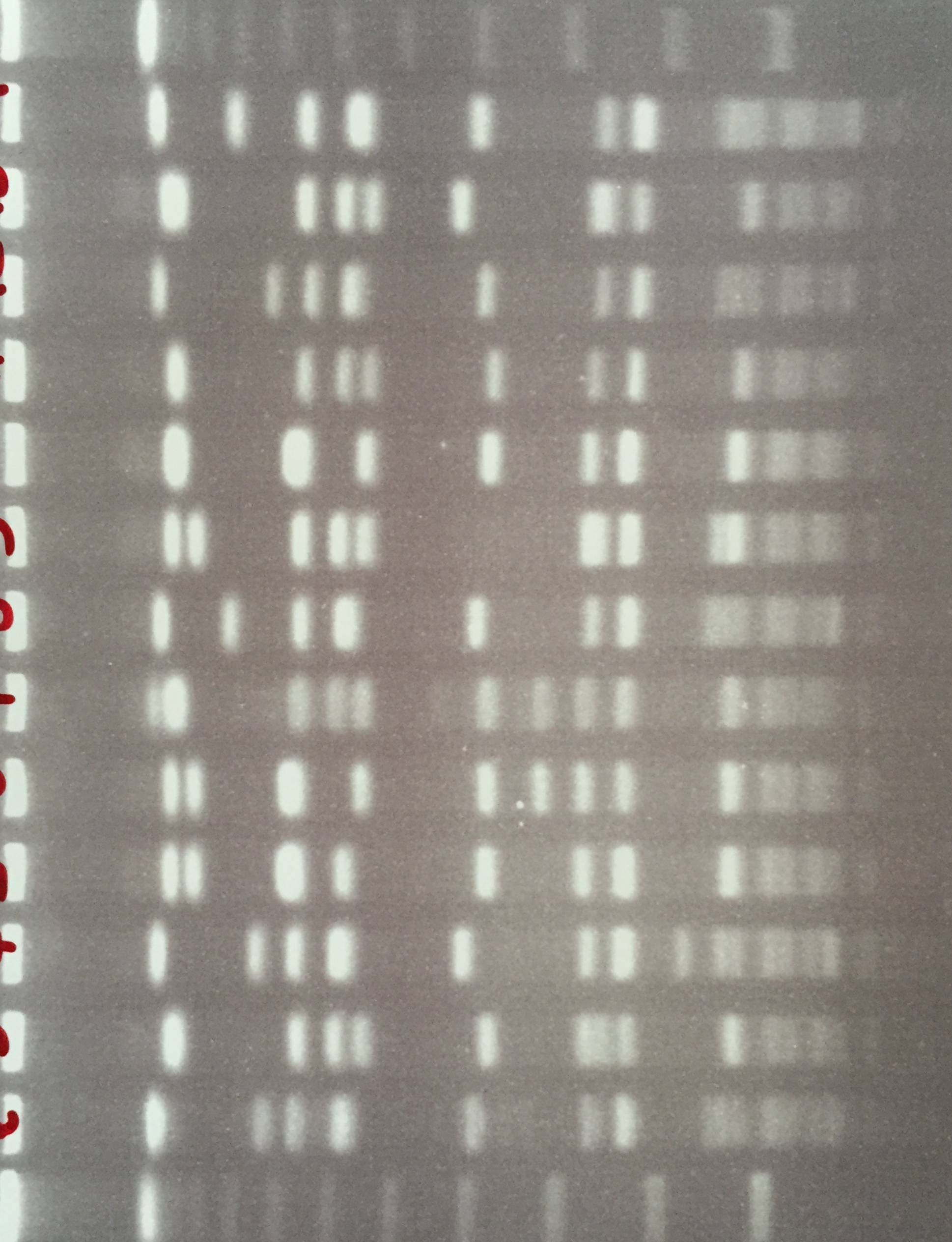 | related |
|  |  |  |  |  |  |
| hci-120 | rc-120 | 6 (20.7) | 163-42 (2) | 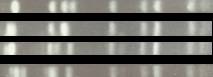 | related |
|  |  |  |  |  |  |

| hci-631 | med-631 | 3 (25.0) | 445-3 (2) | 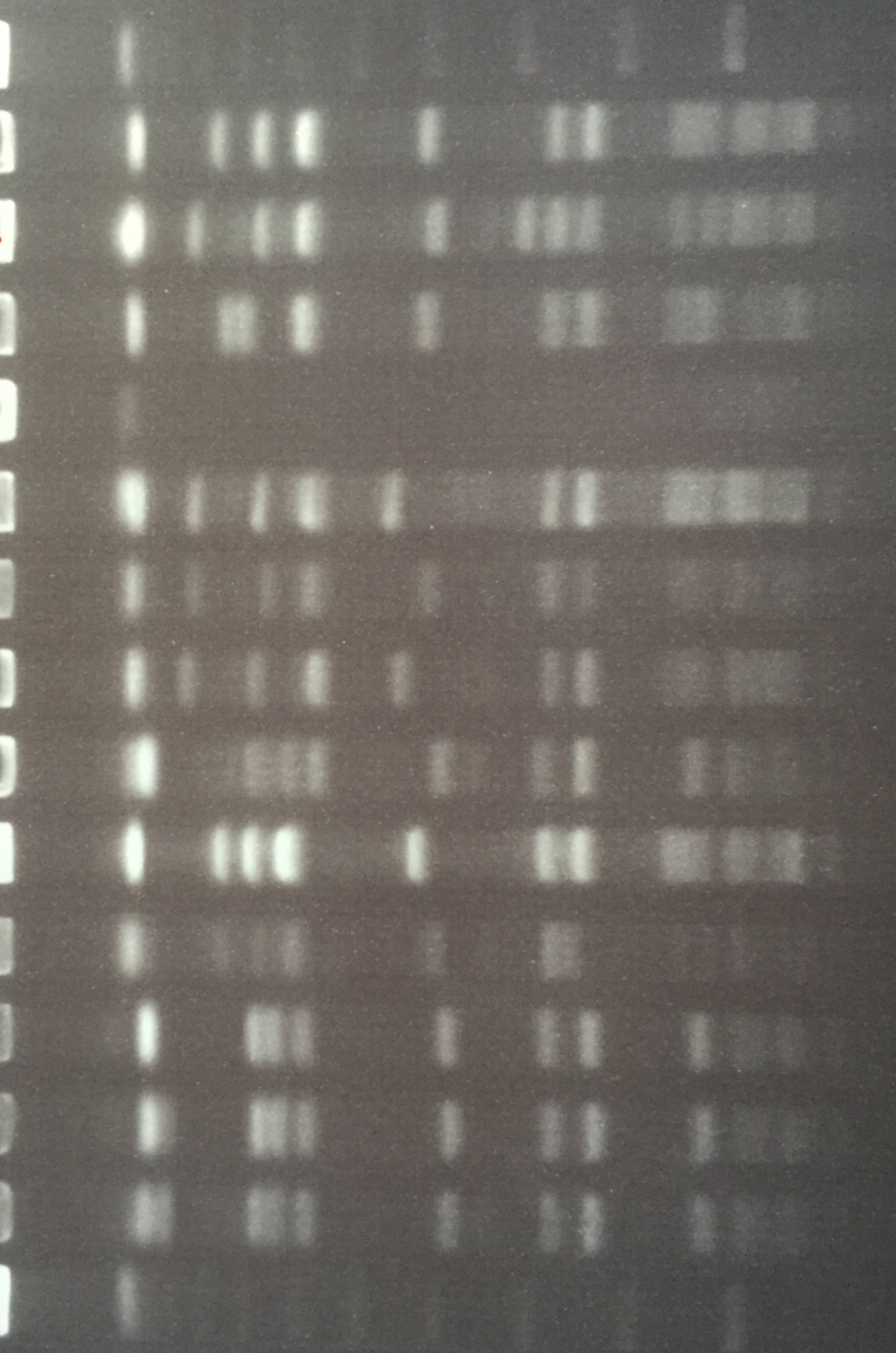 | related |
| --- | --- | --- | --- | --- | --- |
| nh-3154 | nhu-3154 | 5 (25.0) | 854-3 (2) | 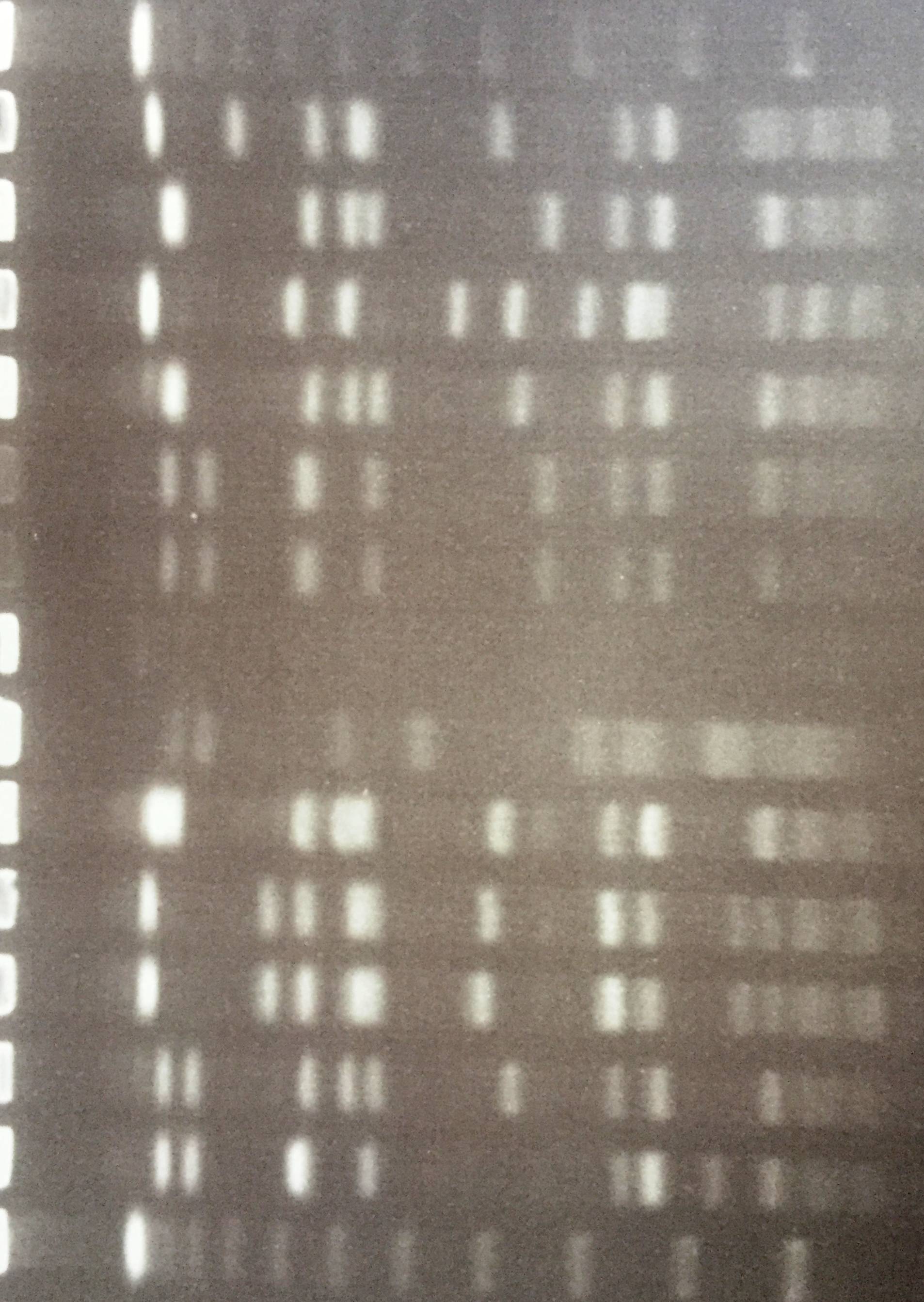 | identical |
